# Does the Swedish Interactive Threshold Algorithm (SITA) accurately map visual field loss attributed to vigabatrin?

**DOI:** 10.1186/1471-2415-14-166

**Published:** 2014-12-23

**Authors:** Miriam L Conway, Sarah L Hosking, Haogang Zhu, Robert P Cubbidge

**Affiliations:** Department of Optometry and Visual Science, City University London, Northampton Square, London, EC1V OHB UK; Department of Optometry University of Melbourne, University of Melbourne, 32 Gisborne Street, East Melbourne, VIC 3002 Australia; Optometry & Vision Sciences, School of Life & Health Sciences, Aston University, Aston Triangle, Birmingham, B4 7ET UK

**Keywords:** Swedish interactive threshold algorithm, Vigabatrin, Visual field

## Abstract

**Background:**

Vigabatrin (VGB) is an anti-epileptic medication which has been linked to peripheral constriction of the visual field. Documenting the natural history associated with continued VGB exposure is important when making decisions about the risk and benefits associated with the treatment. Due to its speed the Swedish Interactive Threshold Algorithm (SITA) has become the algorithm of choice when carrying out Full Threshold automated static perimetry. SITA uses prior distributions of normal and glaucomatous visual field behaviour to estimate threshold sensitivity. As the abnormal model is based on glaucomatous behaviour this algorithm has not been validated for VGB recipients. We aim to assess the clinical utility of the SITA algorithm for accurately mapping VGB attributed field loss.

**Methods:**

The sample comprised one randomly selected eye of 16 patients diagnosed with epilepsy, exposed to VGB therapy. A clinical diagnosis of VGB attributed visual field loss was documented in 44% of the group. The mean age was 39.3 years ± 14.5 years and the mean deviation was -4.76 dB ±4.34 dB. Each patient was examined with the Full Threshold, SITA Standard and SITA Fast algorithm.

**Results:**

SITA Standard was on average approximately twice as fast (7.6 minutes) and SITA Fast approximately 3 times as fast (4.7 minutes) as examinations completed using the Full Threshold algorithm (15.8 minutes). In the clinical environment, the visual field outcome with both SITA algorithms was equivalent to visual field examination using the Full Threshold algorithm in terms of visual inspection of the grey scale plots , defect area and defect severity.

**Conclusions:**

Our research shows that both SITA algorithms are able to accurately map visual field loss attributed to VGB. As patients diagnosed with epilepsy are often vulnerable to fatigue, the time saving offered by SITA Fast means that this algorithm has a significant advantage for use with VGB recipients.

**Electronic supplementary material:**

The online version of this article (doi:10.1186/1471-2415-14-166) contains supplementary material, which is available to authorized users.

## Background

Vigabatrin (VGB) is the first purposely designed anti-epileptic medication. It is administered as a first line therapy to patients suffering from Infantile Spasms and as an adjunctive therapy to patients with complex partial seizures [1, 2]. VGB was initially approved for use within the United Kingdom and several other European countries in 1989 however, it is now well established that VGB therapy can induce peripheral constriction of the visual field. Reports of the prevalence of this defect range between 17% [3] and 86% [4]. The risk of VGB attributed field loss appears to be lower in children less than 12 years old exposed to VGB [5] when compared to adults and adolescents who receive VGB at a later age [6]. General consensus suggests that the visual field loss is permanent [7, 8]. Positive relationships between the visual field loss and cumulative dosage [3, 8–10] duration of vigabatrin therapy [6, 8–11] and maximum daily dosage [12] have all been documented. Despite these known side effects, in the last few years VGB has become licensed in Canada, Mexico and the United States for the same uses as reported above.

Patients receiving VGB are frequently monitored using either kinetic perimetry or automated static perimetry [13]. Vigabatrin attributed visual field loss is two and a half times more common with automated static perimetry when compared to manual kinetic perimetry [14]. The authors attributed these findings to the fact that in general, static perimetry is more sensitive than kinetic perimetry for the detection of field loss [15]. Static perimetry is normally carried out using the Swedish Interactive Threshold Algorithms (SITA). These algorithms were developed with the specific aim of offering a significant reduction in examination time but without sacrificing any loss in the accuracy of threshold estimation when compared to the Full Threshold and FASTPAC algorithms [16, 17].

SITA is based on prior distributions of normal and abnormal visual field behaviour to estimate the threshold. The prior distribution models contain information on age-corrected normal threshold values, frequency-of-seeing curves, between-subject variability and inter-point correlations between thresholds [16]. The abnormal visual field model is based on a glaucomatous population and thus inter-point correlations of threshold values would be based upon the retinal nerve fibre arrangement [18]. VGB induced visual field loss however is thought to result from a toxic effect in the retina and as such may not precisely conform to the nerve fibre bundle. Consequently, SITA may map a response for VGB recipients which are artificially influenced by the glaucomatous model.

To date, the only study to have examined whether SITA was valid in patients with non-glaucomatous pathologies, such as optic neuropathies and hemianopias, reported that SITA Standard was at least as good as Full Threshold for the detection of visual loss in individual examinations [19]. For VGB recipients it is essential that we accurately document the natural progression associated with continued VGB exposure. This information is vital when making decisions on management as it allows the patient and practitioner an opportunity to weigh up the risks and benefits associated with continued treatment.

The aim of this investigation was to assess the clinical utility of the SITA algorithms for the investigation of patients receiving VGB. Firstly, by determining the threshold agreement between the different algorithms. Secondly, by determining the threshold agreement within the same algorithm across two successive visits. Thirdly, the agreement of the diagnostic outcome was assessed in terms of area and depth of defect.

## Methods

Twenty-Two participants: 12 females and 10 males (mean age 38.54 years, SD 13.45, range 16 to 61 years) who were undergoing or who had previously undergone treatment with VGB were invited to take part in the study. Four participants were removed from the study as they had a visual field defect not attributed to VGB another two participants were removed because of poor reliability. The sample therefore consisted of 16 epilepsy patients; 10 females and 6 males (mean age 39.3, SD 14.52, range 18 to 61). Patients were recruited from National Health Service hospitals in the United Kingdom and written informed consent was obtained from each participant prior to commencement of the study. Approval for the study was given by the Aston University Human Sciences Ethical Committee and adhered to the tenets of the Declaration of Helsinki.

Vigabatrin recipients had their visual field measured using both SITA strategies and the Full Threshold algorithm. The Full Threshold algorithm does not use prior distributions of normal and abnormal visual field behaviour to estimate threshold sensitivity but instead employs a 4-2 dB staircase to estimate each threshold sensitivity. The design of the Full Threshold algorithm ensures that the visual field is not artificially influenced by prior models and therefore provided the gold standard for mapping VGB attributed field loss. At the first visit, all patients underwent a 30-2 visual field examination on both eyes using the Full Threshold algorithm (Humphrey Field Analyser 750 software version A10.2). This visit served to reduce the learning effect observed in perimetry [20] and the results were not used for data analysis. At the second and third visits, each patient underwent perimetry on one randomly assigned eye which remained constant for a given patient according to one of four randomly assigned protocols (Table 1). This unconventional order protocol was designed to induce similar degrees of fatigue within all three algorithms by ensuring that the first and second test sessions were of similar duration.

**Table 1:** **The test order sequence randomly assigned to patients**

Protocol	Test order sequence for visit 2 and visit 3		
	**First Session**	**Rest period**	**Second Session**
**A**	Full Threshold	30 minute rest period	SITA Standard then SITA Fast
**B**	Full Threshold	30 minute rest period	SITA Fast then SITA Standard
**C**	SITA Standard then SITA Fast	30 minute rest period	Full Threshold
**D**	SITA Fast then SITA Standard	30 minute rest period	Full Threshold

Visual fields obtained from the left eye were changed to the right eye format and the stimulus locations immediately above and below the blind spot were removed from the analysis which was twofold. Firstly, threshold data was evaluated in terms of threshold agreement between algorithms (during visit 3) and threshold agreement within algorithm (between visit 2 and 3). The second analysis was in terms of the clinical status of the visual field both within and between threshold algorithms.

## Results

The sample comprised a typical cross section of VGB therapy patients (Table 2). Forty four percent of the patients had a confirmed clinical diagnosis of VGB attributed field loss from their medical records. The diagnosis was made independently from the research study for purely clinical purposes. Visual field defects exhibited a bilateral symmetrical defect showing concentric constriction of the peripheral visual field which was more pronounced nasally and typically characteristic of VGB attributed field loss. The other 66% of the sample had a confirmed clinical diagnosis of no visual field loss from their medical records. All patients had a visual acuity of 6/9 or better and ametropia not exceeding ±5.00 day and ±2.50 day of astigmatism. No patient had a history of ocular disease or previous surgery to their eye or brain. All visual fields fell inside the criteria of less than 33% false positive, less than or equal to 33% false negative and 20% fixation losses (see Additional file 1: Table S1). We acknowledge that the false negative rate was higher for the patients with significant visual field loss. However, it is now well recognised in perimetry that the false negative catch trial methods are not suitable for estimating patient attentiveness in eyes with significant visual field loss visual field loss as the frequency of false-negative responses in eyes with visual field defects is associated with amount of field loss [21].

**Table 2:** **The seizure history of the patient group and the epileptic medications concomitant with vigabatrin**

Patient number	Seizure history	Carbamazepine	Sodium valporate	Clobazam	Levetiracetam	Topiramate	Lamotrogine	Phenytoin	Gabapentin	Other
1	Unknown							X		X
2	generalised	X	X	X			X		X	X
3	Complex partial partial/secondary generalised						X			X
4	Complex partial									
5	Simple/complex		X		X					
6	Complex partial	X							X	
7	Complex partial	X			X					
8	Unknown	X				X				
9	Generalised						X			
10	Complex partial	X			X					
11	Complex partial			X			X			
12	Unknown	X								
13	Generalised	X	X				X	X		X
14	Complex partial partial/secondary generalised				X					
15	Unknown	X	X					X		
16	Complex partial partial/secondary generalised			X			X		X	

### Threshold agreement between and within algorithms

One-way analysis of variance (between-subject) was used to determine whether there was any significant difference between examination duration, mean sensitivity (MS) mean deviation (MD) and pattern standard deviation (PSD), in any of the three algorithms resulting from the various sequence options of perimetric examination. There was no significant difference between protocol and visual field index at the third visit indicating that the unconventional order protocol did not influence threshold sensitivities. The group mean values for MS, MD, PSD and test duration (±1 SD) for each algorithm at the second and third visits are illustrated in Table 3.

**Table 3:** **Group global indices and examination times**

		Full threshold	SITA standard	SITA fast
**Mean sensitivity (dB)**	**Visit 2**	24.30 *(5.28)*	25.30 *(5.66)*	26.14 *(5.36)*
	**Visit 3**	24.05 *(5.04)*	25.30 *(6.29)*	25.56 *(5.34)*
**Mean deviation (dB)**	**Visit 2**	-4.24 *(4.00)*	-4.25 *(4.46)*	-3.90 *(4.76)*
	**Visit 3**	-4.89 *(3.79)*	-5.06 *(4.98)*	-4.34 *(4.25)*
**Pattern standard deviation (dB)**	**Visit 2**	4.70 *(3.67)*	4.78 *(3.91)*	3.99 *(3.30)*
	**Visit 3**	4.89 *(3.51)*	4.95 *(3.97)*	4.63 *(3.64)*
**Examination time (seconds)**	**Visit 2**	937.0 *(157.9)*	449.0 *(80.9)*	279.4 *(79.2)*
	**Visit 3**	956.6 *(165.1)*	464.1 *(99.4)*	282.3 *(67.3)*

For MD and PSD, there was no difference between algorithms or visit order. The mean sensitivity of SITA Standard was 1.25 dB higher and 1.51 dB higher for the SITA Fast algorithm at the final visit compared to the Full Threshold algorithm SITA Standard (7 .6 minutes) and SITA Fast (4.7 minutes) were significantly faster than Full Threshold perimetry (15.8 minutes).

**Figure 1:**
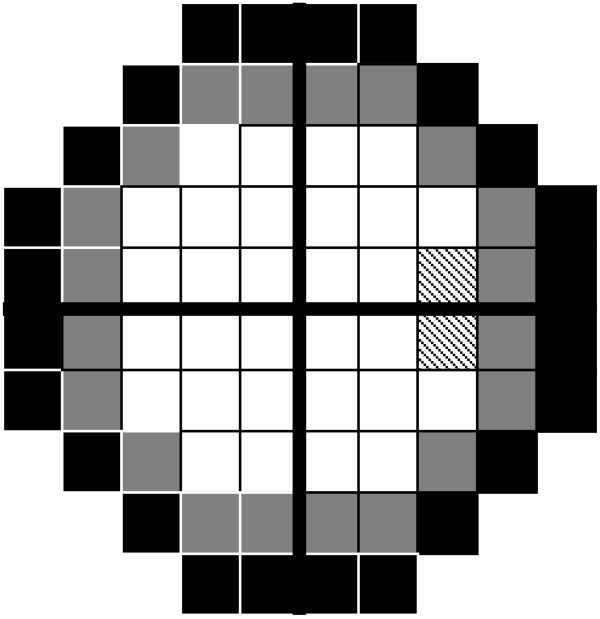
**Illustration of outer (black), middle (grey) and inner (white) sectors (blind spot dashed lines).**

**Figure 2:**
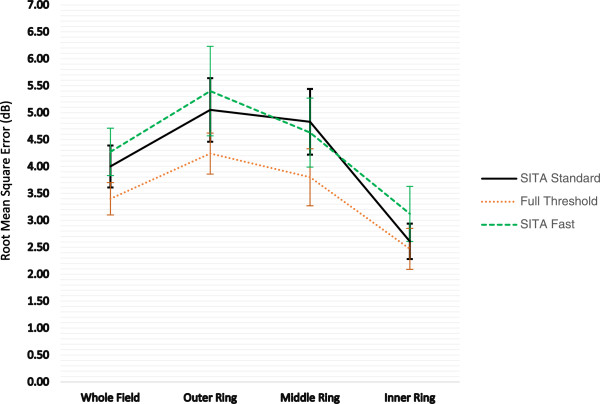
**Root Mean Square Error (dB) and 95% Confidence Intervals as a function of algorithm for the whole field, outer ring, middle ring and inner ring.**

The threshold agreement within each algorithm across 2 successive visits was assessed by calculating the root mean square error (RMSE) for all test locations, participants and algorithms. The visual field was sectorised into outer, middle and inner zones of eccentricity; the outer zone comprised of 24 points from 25.8 to 28.5 degrees from fixation, the middle zone comprised 20 stimulus locations from 21.2 to 22.8 degrees from fixation and the inner zone comprised of 30 stimulus locations from 4.2 to 17.5 degrees from fixation (Figure 1). Figure 2 shows the 95% confidence intervals for the RMSE as a function of threshold algorithm for the whole field and the outer, middle and inner visual field regions. Within algorithm threshold variability was lowest in the inner ring for all threshold algorithms. As sectors increase in eccentricity from fixation, the group mean RMSE increased indicating less threshold agreement within an algorithm across visits 2 and 3. Both SITA algorithms had less threshold agreement (group mean RMSE) and larger confidence intervals across all visual field regions when compared to Full Threshold (Figure 2). Within the outer field region the RMSE with SITA Fast was 27% higher than Full Threshold and SITA Standard was 19% higher than Full Threshold (p = 0.017). Similar differences were found across all field regions.

### Clinical status of the visual field

**Figure 3:**
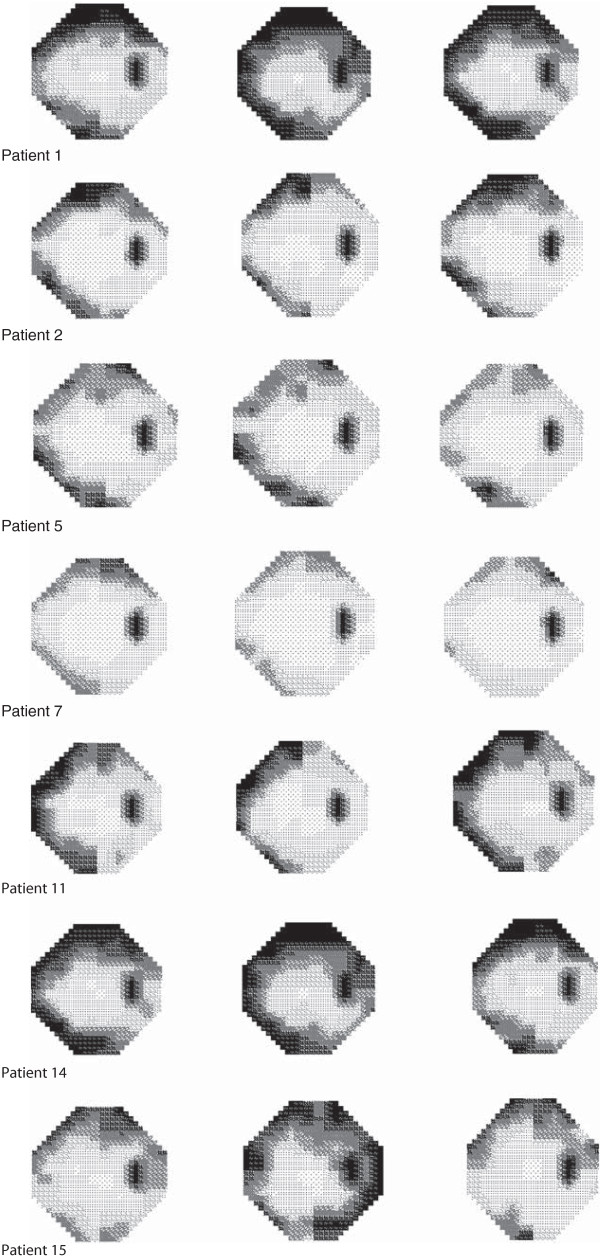
**Grey Scale plots for all patients with a VGB attributed visual field defect: left Full Threshold, middle SITA Standard, right SITA Fast.**

All visual fields were categorised using the classification defined by Wild et al. (2009) [22]. Visual defects ranged from mild to severe (Table 4) based on the number and position of stimulus locations exhibiting an abnormality at either p < 0.01 or p < 0.005 out to 30 degrees eccentricity for static threshold perimetry and were present in 38% after Full threshold was assigned the gold standard for the detection of VGB attributed visual field loss. The false positive and false negative rate was then calculated for both SITA algorithms. False positive rate was defined as the proportion of persons falsely identified as diseased persons by SITA Standard or SITA Fast in those without any VGB attributed field loss identified by the gold standard Full Threshold algorithm. One patient was falsely diagnosed as having VGB attributed visual field loss with both SITA algorithms when there was none documented on Full Threshold (1/10 = 10%) suggesting that the false positive rate was 10% for both SITA algorithms. Closer inspection of the “normal” Full Threshold visual field plot (patient 15 Figure 3) reveals that if those locations demonstrating a 2% loss on pattern probability analysis were also included in the analysis then the patient would have been diagnosed with a VGB attributed defect. Additionally, information from their medical records shows that this patient had a confirmed clinical diagnosis of VGB attributed field loss from previous visual field testing. The false negative rate was defined as the proportion of persons falsely identified as normal by SITA Standard or SITA Fast, among people with VGB attributed field loss identified by the gold standard Full Threshold algorithm. SITA standard correctly identified all patients with VGB attributed field loss documented on Full Threshold as having VGB attributed field loss on SITA standard suggesting a false negative rate of 0%. One patient was falsely identified as being normal with SITA Fast when there was a VGB attributed defect on the Full Threshold algorithm (1/6) suggesting a false negative rate of 17%. Closer inspection of the falsely identified field (patient 7 Figure 3) reveals that if those locations demonstrating a 2% loss on shape probability analysis were also included in the analysis then the patient would have been correctly diagnosed.

**Table 4:** **Severity classification for all patients categorised using criteria by Wild et al. (2009)** [19]

Patient no.	Full threshold	SITA standard	SITA fast
	**Classification**	**Classification**	**Classification**
1	Severe	Severe	Severe
2	Moderate	Moderate	Moderate
3	Nil	Nil	Nil
4	Nil	Nil	Nil
5	Moderate	Moderate	Moderate
6	Nil	Nil	Nil
7	Mild	Mild	Nil
8	Nil	Nil	Nil
9	Nil	Nil	Nil
10	Nil	Nil	Nil
11	Severe	Severe	Severe
12	Nil	Nil	Nil
13	Nil	Nil	Nil
14	Severe	Severe	Severe
15	Nil	Severe	Moderate
16	Nil	Nil	Nil

To further assess the similarity in area of any visual field defect mapped by each threshold algorithm the number of non-overlapping defects or normal locations between two visual fields i.e. the dissimilarity between the fields was calculated and expressed as a percentage of the total number of visual field locations. Comparisons were made between threshold algorithms at visit 3 and also within each algorithm between visits 2 and 3. The group mean differences are illustrated in Table 5.

**Table 5:** **Group mean percentage of dissimilar total probability stimulus locations classified as normal or defective (±1 SD) between threshold algorithms at the final visit (top) and between examinations using the same threshold algorithm (bottom)**

	**Percentage difference in defect area between threshold algorithm**		
	**(at Visit 3)**		
	**Full Threshold - SITA Standard**	**Full Threshold - SITA Fast**	**SITA Standard - SITA Fast**
**Total deviation**	16.2 *(16.8)*	17.1 *(17.9)*	17.1 *(17.5)*
	**Percentage difference in defect area within threshold algorithm**		
	**(between Visits 2 and 3)**		
	**Full Threshold**	**SITA Standard**	**SITA Fast**
**Total deviation**	14.0 *(14.2)*	13.0 *(13.3)*	19.6 *(20.5)*

For total deviation probabilities, the average area of visual field defect between two threshold algorithms at the final visit varied by up to 17%. Although the least variation was demonstrated between Full Threshold and SITA Standard none of the differences were found to be statistically significant. Using a given threshold algorithm the average area of visual field defect varied by a maximum of 20% of stimulus locations between the second and third visits. Although SITA Fast yielded the greatest difference there was no statistical difference in this variability between threshold algorithms.

In order to demonstrate how each SITA algorithm displays the spatial pattern of VGB attributed field loss. The grey scale plots from all 3 testing strategies are presented for every patient with confirmed VGB attributed field loss (Figure 3) and also those without visual field loss (Additional file 2: Figure S1). Visual field plots were generated from the Humphrey sensitivity values (dB) using software which is freely available from one of the authors (HZ) [23, 24]. Visual inspection of the plots shows they were approximately equivalent, for all 3 algorithms, for every patient with and without a confirmed VGB attributed visual field defect.

**Figure 4:**
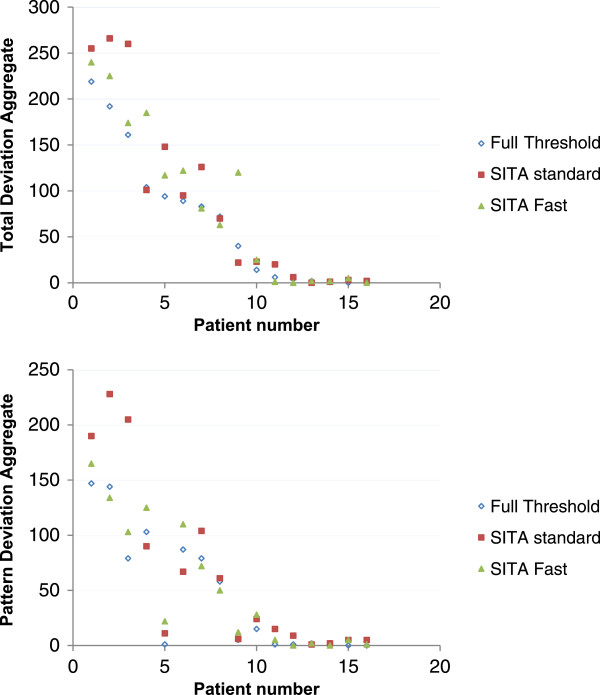
**Showing the sum of total deviation aggregate (Top) and pattern deviation aggregate (bottom) probability levels for every patient.**

In order to analyse differences between algorithms in estimating defect depth, each stimulus location was given a numerical value corresponding to the level of total or pattern deviation significance (0 = not significant, 1 = 5%, 2 = 2%, 3 = 1%, 4 = 0.5%). For every patient the sum of all visual field locations was calculated for each algorithm for both total and pattern deviation probability plots (Figure 4). A repeated measures analysis of variance was conducted to explore any differences in estimating defect depth across all three algorithms for either total or pattern deviation probability plots. As you can see from Figure 4 overall the SITA Standard algorithm produced the most severe visual field loss, for each patient followed by SITA Fast and Full Threshold. These small differences however did not reach statistical significance between algorithms for either total deviation or the pattern deviation probability plots.

## Discussion

The findings that both SITA Standard was on average approximately twice as fast (7.6 minutes) and SITA Fast approximately 3 times as fast (4.7 minutes) as examinations completed using the Full Threshold algorithm (15.8 minutes) is in agreement with other studies of SITA in the normal population [25, 26] in the investigation of glaucoma [27] and neuro-ophthalmological conditions [19] and adds nothing new to the existing literature. Test duration is of particular importance when examining patients with epilepsy as they are more prone to fatigue than other patient groups [28]. Researchers have shown that approximately 25% of patients (n = 734) with epilepsy are unable to produce a conclusive visual field test at any visit [14]. This finding suggests that examination speed is particularly important in this group of patients and has led the authors to suggest that ocular imaging of the retinal nerve fibre layer [29, 30] might be a useful technique for those patients who are unable to carry out perimetry. Based on examination times our results suggest that SITA Fast would be the obvious choice of algorithm however it is vital that the algorithm of choice is able to yield both a high threshold agreement and a comparable diagnostic outcome equivalent to the Full Threshold algorithm.

Compared to the Full Threshold algorithm, the group mean pointwise sensitivities were on average 1.1 dB greater for SITA Standard and 1.7 dB greater for SITA Fast (Table 3). These results are concordant with findings in both normal and glaucomatous populations which report higher sensitivity as a function of algorithm in the order Full Threshold < SITA Standard < SITA Fast [31]. The higher sensitivity of SITA compared to the Full Threshold algorithm would be expected in SITA as the threshold is defined as the stimulus intensity which has a probability of seeing of 50% whereas in Full Threshold the estimate is the last seen stimulus using a step size of 2 dB [32]. Differences between SITA types could be explained by the greater imprecision in threshold estimate of SITA Fast which is integral to the design of the algorithm [16].

When considering the global indices MD and PSD there was no significant difference between any of the algorithms which suggests that they are interchangeable as diagnostic algorithms. However, global measures of the visual field status do not give a true representation of the nature and depth of visual field loss and do not yield any spatial information relating to the extent of visual field loss. Thus, it is necessary to examine the pointwise differences in sensitivity between and within threshold algorithms.

Threshold agreement within and between algorithms when defined by the RMSE was assessed for the whole visual field and in the concentric outer, middle and inner rings (Figure 1). Each algorithm yielded the smallest group RMSE within the inner rings and the greatest RMSE for the outer ring. This finding is unsurprising as the inner visual field ring is relatively spared by VGB toxicity and predominantly yields thresholds which are considered within the normal range. Furthermore, normal threshold variability increases with eccentricity which is reflected in greater confidence intervals in the visual field periphery [18]. As the frequency of defect in VGB toxicity is greatest in the outer ring [12], the magnitude of the RMSE is also greater because this visual field area is damaged and thus contains a wider range of threshold values. Both SITA algorithms had less threshold agreement (group mean RMSE) and larger confidence intervals across all visual field regions when compared against Full Threshold (Figure 2). Within the outer field region the RMSE with SITA Fast was 27% higher than Full Threshold and SITA Standard was 19% higher than Full Threshold. Similar differences were found across all field regions suggesting that the SITA algorithms are equivalent methods for quantifying VGB attributed field loss. Visual fields are often unreliable in patients diagnosed with epilepsy [6]. When evaluating serial visual field tests researchers have reported that VGB recipients often show a variable degree of “normal” fluctuation that is not related to the pathological damage itself [33]. Both SITA algorithms demonstrated slightly less threshold agreement and larger between-subject variability when compared against the Full threshold algorithm (Figure 2). This might be because the faster paced SITA algorithms might be slightly more difficult in patients diagnosed with epilepsy either because of the aetiology associated with the epilepsy itself or because of the combination of medications that they are receiving.

Diagnostic outcome agreement was assessed in terms of false positive and false negative rate, area of defect, grey scale plots and depth of the defect. Results have indicated that the false negative rate was 0% for SITA Standard and 17% for SITA Fast. Closer inspection of the one falsely identified field reveals that if those locations which demonstrated a 2% loss on shape probability analysis were also included in the analysis then the patient would also have been correctly diagnosed as having VGB attributed field loss using SITA Fast. The false positive rate of both SITA strategies was 10% (n = 1). Further inspection of the “normal” visual field documented with Full Threshold algorithm however suggests a borderline visual field defect suggestive of VGB toxicity. Additionally, information from the patient’s medical records confirmed a clinical diagnosis of a VGB attributed field loss on previous visual field testing. This finding suggests that both SITA were correct in their diagnosis of a VGB attributed defect and the false positive rate was therefore 0%.

On average, in terms of defect area there was a lack of coincidence of the defect at approximately 17% of stimulus locations (total deviation probability). This inconsistency may reflect the normal physiological and psychological factors which influence perimetric examination [20, 34]. When one threshold algorithm was compared to another, the level of probability defining the defect depth showed little difference across all algorithms. Whilst SITA Standard gave comparable results to the Full Threshold algorithm in terms of the defect depth at successive examinations, SITA Fast showed slightly less agreement. However, this difference was small and there was no statistical difference in this variability between threshold algorithms consequently this factor is not expected to confound clinical diagnosis. Results are in agreement with a visual inspection of the grey scale plots which reveals that all 3 algorithms produce equivalent field loss in relation to their grey scale plots (Figure 3). Overall SITA Standard algorithm appeared to show the greatest severity of visual field loss, for each patient followed by SITA Fast and Full Threshold (Figure 4). These small differences however again did not reach statistical significance between algorithms for either total deviation or the pattern deviation probability plots.

Our research was able to detect differences between algorithms on both a global and regional level suggesting that the sample size was large enough to detect these subtle differences. However like most studies we acknowledge that our study would probably benefit from replication. We have demonstrated that the SITA threshold modelling procedure is capable of mapping the nature of VGB induced visual field loss in terms of its area and depth. In the ideal clinical environment the optimal threshold algorithm used in perimetry should be fast but able to accurately estimate the threshold consistently across successive examinations. Until the arrival of SITA there was always a trade off between reducing speed of examination and increasing variability in the threshold which had implications for both initial diagnosis and monitoring of visual field loss over time.

In the clinical environment, the visual field outcome with both SITA algorithms was directly equivalent to visual field examination using the Full Threshold algorithm in terms of defect area, defect severity and visual inspection of the grey scale plot. SITA was not comparable when threshold agreement within each algorithm (RMSE) was calculated between Full Threshold and the SITA algorithms for the outer ring (p = 0.017). When we analyzed how these small differences in threshold agreement transferred into the number of non-overlapping defects or normal locations between two visual fields within the same algorithm. We found little variation suggesting that these differences have little clinical impact.

The only other study to have evaluated SITA’s efficacy in detecting eye disease other than glaucoma concluded that SITA Standard was at least as good as the Full Threshold in detecting both optic neuropathy and hemianopic visual field loss [19]. The authors were unable to comment on SITA’s efficacy in examining these pathologies across visits as they did not carry out serial visual field tests.

## Conclusion

Our research shows that both SITA algorithms are able to accurately map visual loss attributed to VGB across 2 successive examinations. Our findings in conjunction with the knowledge that patients diagnosed with epilepsy are particularly vulnerable to fatigue [28] suggest that SITA Fast might have an advantage when testing this particular group of patients.

## Electronic supplementary material

Additional file 1: Table S1: Reliability criteria for all patients (FT = Full Threshold; SS = SITA Standard; SF = SITA Fast; V2 = visit 2; V3 = Visit 3). (DOCX 14 KB)

Additional file 2: Figure S1: Grey Scale plots for all patients with no visual field defect: left Full Threshold, middle SITA Standard, right SITA Fast. (ZIP 15 MB)
