# Supplementary material for: Does the Swedish Interactive Threshold Algorithm (SITA) accurately map visual field loss attributed to vigabatrin?
Source: BMC Ophthalmol. 2014 Dec 23;14:166. doi: 10.1186/1471-2415-14-166 (PMC4391113; doi:10.1186/1471-2415-14-166)
Supplement: Supplementary file 1 — Additional file 1: Table S1: Reliability criteria for all patients (FT = Full Threshold; SS = SITA Standard; SF = SITA Fast; V2 = visit 2; V3 = Visit 3). (DOCX 14 KB) [file 12886_2013_550_MOESM1_ESM.docx]

| Patient no. | FT Fixation losses V2 | FT False positive V2 | FT False  Negative V2 | FT Fixation losses V3 | FT False positive  V3 | FT False  Negative  V3 | SS Fixation losses V2 | SS False positive  V2 | SS False  Negative  V2 | SS Fixation losses  V3 | SS False positive  V3 | SS False  Negative  V3 | SF Fixation losses  V2 | SF False positive  V2 | SF False  Negative  V2 | SF Fixation losses  V3 | SF False positive  V3 | SF False  Negative  V3 |
| --- | --- | --- | --- | --- | --- | --- | --- | --- | --- | --- | --- | --- | --- | --- | --- | --- | --- | --- |
| 1 | 0% | 0% | 18% | 7% | 0% | 21% | 0% | 3% | 21% | 0% | 4% | 6% | 13% | 3% | 33% | 0% | 2% | 33% |
| 2 | 3% | 0% | 5% | 8% | 5% | 0% | 0% | 0% | 8% | 5% | 1% | 8% | 0% | 0% | 14% | 6% | 2% | 16% |
| 3 | 0% | 0% | 0% | 0% | 0% | 0% | 0% | 0% | 0% | 0% | 2% | 6% | 0% | 0% | 2% | 0% | 0% | 0% |
| 4 | 4% | 7% | 0% | 9% | 14% | 7% | 0% | 0% | 0% | 0% | 1% | 0% | 25% | 0% | 0% | 8% | 0% | 0% |
| 5 | 3% | 0% | 0% | 0% | 0% | 6% | 0% | 0% | 7% | 0% | 2% | 10% | 7% | 0% | 0% | 0% | 0% | 11% |
| 6 | 0% | 0% | 0% | 0% | 0% | 0% | 6% | 1% | 0% | 0% | 0% | 2% | 0% | 0% | 0% | 0% | 2% | 3% |
| 7 | 4% | 0% | 0% | 0% | 0% | 0% | 11% | 1% | 0% | 5% | 0% | 0% | 7% | 0% | 11% | 8% | 0% | 5% |
| 8 | 0% | 0% | 0% | 4% | 7% | 0% | 13% | 12% | 0% | 5% | 0% | 6% | 17% | 7% | 0% | 0% | 3% | 10% |
| 9 | 0% | 0% | 0% | 5% | 0% | 0% | 0% | 0% | 0% | 6% | 1% | 1% | 0% | 0% | 0% | 14% | 0% | 0% |
| 10 | 0% | 0% | 7% | 4% | 0% | 0% | 0% | 7% | 7% | 6% | 5% | 7% | 8% | 5% | 1% | 8% | 8% | 15% |
| 11 | 0% | 0% | 18% | 0% | 0% | 12% | 0% | 1% | 12% | 0% | 0% | 10% | 0% | 1% | 22% | 0% | 0% | 8% |
| 12 | 0% | 0% | 0% | 0% | 0% | 0% | 0% | 0% | 0% | 0% | 1% | 2% | 15% | 1% | 1% | 0% | 0% | 0% |
| 13 | 0% | 0% | 0% | 0% | 0% | 0% | 6% | 0% | 0% | 0% | 0% | 0% | 0% | 0% | 1% | 0% | 0% | 1% |
| 14 | 3% | 0% | 32% | 0% | 0% | 5% | 0% | 0% | 20% | 5% | 0% | 6% | 7% | 4% | 20% | 0% | 0% | 0% |
| 15 | 4% | 0% | 11% | 0% | 0% | 10% | 0% | 0% | 10% | 1% | 4% | 26% | 0% | 0% | 6% | 0% | 0% | 13% |
| 16 | 7% | 0% | 0% | 18% | 0% | 0% | 11% | 2% | 5% | 18% | 2% | 0% | 0% | 0% | 4% | 8% | 4% | 10% |

**Additional file 1: Table S1**: Reliability criteria for all patients (FT= Full Threshold; SS = SITA Standard; SF = SITA Fast; V2 = visit 2; V3 = Visit 3)
