# Supplementary material for: Does the Swedish Interactive Threshold Algorithm (SITA) accurately map visual field loss attributed to vigabatrin?
Source: BMC Ophthalmol. 2014 Dec 23;14:166. doi: 10.1186/1471-2415-14-166 (PMC4391113; doi:10.1186/1471-2415-14-166)
Supplement: Supplementary file 2 — Additional file 2: Figure S1: Grey Scale plots for all patients with no visual field defect: left Full Threshold, middle SITA Standard, right SITA Fast. (ZIP 15 MB) [file 12886_2013_550_MOESM2_ESM.zip › 2511870061089548_add4.docx]

Patient 12









Patient 16

Patient 13
